# Rituximab Associated Hypogammaglobulinemia in Autoimmune Disease

**DOI:** 10.3389/fimmu.2021.671503

**Published:** 2021-05-12

**Authors:** Joanna Tieu, Rona M. Smith, Seerapani Gopaluni, Dinakantha S. Kumararatne, Mark McClure, Ania Manson, Sally Houghton, David R. W. Jayne

**Affiliations:** ^1^ Department of Medicine, University of Cambridge, Cambridge, United Kingdom; ^2^ Vasculitis and Lupus Clinic, Cambridge University Hospitals National Health Service (NHS) Foundation Trust, Cambridge, United Kingdom; ^3^ Adelaide Medical School, University of Adelaide, Adelaide, SA, Australia; ^4^ Clinical Immunology Unit, Cambridge University Hospitals NHS Trust, Cambridge, United Kingdom

**Keywords:** rituximab, hypogammaglobulinemia, autoimmune disease, immunoglobulin replacement therapy, B-cell

## Abstract

**Objective:**

To evaluate the characteristics of patients with autoimmune disease with hypogammaglobulinemia following rituximab (RTX) and describe their long-term outcomes, including those who commenced immunoglobulin replacement therapy.

**Methods:**

Patients received RTX for autoimmune disease between 2003 and 2012 with immunoglobulin G (IgG) <7g/L were included in this retrospective series. Hypogammaglobulinemia was classified by nadir IgG subgroups of 5 to <7g/L (mild), 3 to <5g/L (moderate) and <3g/L (severe). Characteristics of patients were compared across subgroups and examined for factors associated with greater likelihood of long term hypogammaglobulinemia or immunoglobulin replacement.

**Results:**

142 patients were included; 101 (71%) had anti-neutrophil cytoplasm antibody (ANCA) associated vasculitis (AAV), 18 (13%) systemic lupus erythematosus (SLE) and 23 (16%) other conditions. Mean follow-up was 97.2 months from first RTX. Hypogammaglobulinemia continued to be identified during long-term follow-up. Median time to IgG <5g/L was 22.5 months. Greater likelihood of moderate hypogammaglobulinemia (IgG <5g/L) and/or use of immunoglobulin replacement therapy at 60 months was observed in patients with prior cyclophosphamide exposure (odds ratio (OR) 3.60 [95% confidence interval (CI) 1.03 – 12.53], glucocorticoid use at 12 months [OR 7.48 (95% CI 1.28 – 43.55], lower nadir IgG within 12 months of RTX commencement [OR 0.68 (95% CI 0.51 – 0.90)] and female sex [OR 8.57 (95% CI 2.07 – 35.43)]. Immunoglobulin replacement was commenced in 29/142 (20%) and associated with reduction in infection rates, but not severe infection rates.

**Conclusion:**

Hypogammaglobulinemia continues to occur in long-term follow-up post-RTX. In patients with recurrent infections, immunoglobulin replacement reduced rates of non-severe infections.

## Introduction

B cell depletion plays a key role in the management of many autoimmune diseases. Rituximab (RTX) is licensed for use in rheumatoid arthritis (RA) and AAV, and clinical trials have evaluated RTX in other autoimmune conditions including SLE. Despite limited evidence of hypogammaglobulinemia in patients receiving RTX in these studies, it has been consistently identified in observational studies of patients with autoimmune disease (1–7).

Lower baseline immunoglobulin G (IgG) levels, including levels within population norms, have been associated with subsequent hypogammaglobulinemia (2, 4, 6, 7). An association between cumulative RTX exposure and hypogammaglobulinemia has not been demonstrated (2–4, 7).

Although there is no universal IgG threshold for hypogammaglobulinemia, the clinical significance of hypogammaglobulinemia lies in the resultant susceptibility to infection. Extrapolated from the treatment of patients with common variable immunodeficiency (CVID), prophylactic antibiotics and immunoglobulin replacement therapy are considered where there is a combination of hypogammaglobulinemia, poor vaccination responses, and recurrent and/or severe infection.

Although a proportion of patients with hypogammaglobulinemia have been identified following RTX therapy for autoimmune disease in several studies, their longer-term outcomes, including the effects of immunoglobulin replacement therapy, remain unclear.

In a previous study, from which this study cohort derives, 135/243 (56%) patients with systemic autoimmune disease treated with RTX developed hypogammaglobulinemia (4). This was classified as mild (5 to <7 g/L) in 72 (53%), moderate (3 to <5 g/L) in 53 (39%) and severe (<3 g/L) in 10 (7%). In this study, we sought to evaluate the long-term outcomes of patients with previously identified hypogammaglobulinemia.

## Objectives

1. To explore the characteristics of patients with autoimmune disease who develop RTX associated hypogammaglobulinemia and their long-term outcomes.
2. To examine the outcomes of patients with autoimmune disease which develop RTX associated hypogammaglobulinemia requiring immunoglobulin replacement therapy.

## Methods

Patients with multi-system autoimmune disease who had received RTX between February 2003 and November 2012 and had an IgG <7 g/L on at least two occasions were included in this single center, retrospective cohort form the Vasculitis and Lupus Clinic, Addenbrooke’s Hospital, Cambridge, United Kingdom. Data were collected until August 2017 or last recorded follow-up. A previous report from this cohort described immunoglobulin outcomes in 243 patients who had received RTX for the treatment of multi-system autoimmune disease up to November 2012 (4). This report includes extended follow-up of 142 patients who met the above inclusion criteria.

Patients received a standard departmental dose of 2x1g a fortnight apart followed by 1g every 6 months for 2 years. Extension of RTX course and shortened treatment regimens occurred when clinically appropriate. At the time of treatment for these patients, biosimilar products were not available. Clinical assessments and laboratory data were typically obtained 6-monthly, prior to each dose of RTX. Interval data, where available, were also collected.

Patients were excluded if paraproteinemia was detected at any time during follow-up. All immunoglobulin results during periods of nephrotic range proteinuria and for 3 months following plasma exchange were excluded from analyses. Patients were categorized by absolute nadir IgG levels, as mild (5 to <7 g/L), moderate (3 to <5 g/L), and severe (<3 g/L). Infection was defined as any presumed or confirmed infection warranting the use of an oral antimicrobial agent. Severe infection was defined as a presumed or confirmed infection requiring an intravenously administered antimicrobial and/or hospital admission.

Data collected on each patient included age at diagnosis, gender, disease diagnosis and manifestations, age, date, and indication for first RTX prescription, cumulative RTX dose, use of immunosuppressive agent(s) pre-RTX, concurrently and post-RTX, prednisolone use at RTX commencement, and at 6 monthly intervals until 24 months post-RTX, infections, mortality, antibiotic prophylaxis and use and duration of immunoglobulin replacement therapy (intravenous or subcutaneous). Prednisolone was the standard oral glucocorticoid prescribed, with equivalent efficacy to prednisone. Laboratory data were collected for each patient from 1 month prior to rituximab to last follow-up, including IgG, IgM and IgA levels, lymphocyte, and neutrophil counts, and CD19, CD4 and CD8 counts. Flow cytometry for lymphocyte subsets were not routine prior to every RTX infusion. Where available, B cell subsets and antibody titers to pneumococcal, haemophilus, varicella, measles, mumps, rubella, and tetanus were collected.

Concurrent immunosuppression was defined as the use of an immunosuppressive agent for at least 6 weeks from RTX commencement, except for cyclophosphamide where any use within the first 6 weeks was included. Post-RTX immunosuppression was defined as use of an immunosuppressive agent at least 6 weeks after RTX commencement, for at least 3 months.

In the setting of hypogammaglobulinemia, immunoglobulin replacement therapy was typically commenced in patients with recurrent and/or severe infections following specialist clinical immunology evaluation. This generally included the assessment of infection rates, and laboratory parameters including lymphocyte subsets and vaccine responses to *Streptococcus pneumoniae* and *Haemophilus influenzae*, and a trial of prophylactic antibiotics. Prophylactic antibiotic choice was individualized where possible; azithromycin was typically used if not available microbiological or antibiotic sensitivity data was available. Intravenous immunoglobulin replacement therapy was commenced, and patients transitioned to self-administered subcutaneous administration where appropriate. Intravenous immunoglobulin was not used for treatment of underlying autoimmune disease in these patients.

In accordance with the UK National Health Service Research Ethics Committee guidelines, ethics approval was not required as this work comprises anonymous retrospective data and all treatment decisions were made prior to our evaluation.

Dichotomous outcomes are summarized as proportions. Continuous outcomes are summarized as mean and standard deviation if normally distributed, otherwise as median and interquartile range. Comparisons of categorical variables across the immunoglobulin categories were analyzed using Somers’ D to assess for the trend across nadir IgG subgroups. Nominal categorical variables were compared using Chi squared tests or Fisher’s exact test as appropriate. Continuous variables have been compared using Kruskall-Wallis tests. In patients receiving immunoglobulin replacement therapy, infection and severe infection rates were compared by Wilcoxon sign ranked tests. Nadir IgG in the first 12 months were used to examine outcome at 60 and 100 months following the first dose of RTX. A multivariable logistic regression model was used to model outcome (IgG <5g/L or on immunoglobulin replacement therapy) at 60 months. Prespecified explanatory variables were included using a step-wise approach. Model fit was assessed using -2log likelihood, Cox & Snell R square and Nagelkerke R square values. Statistical analyses were performed in SPSS version 24 and figures were produced using Graphpad prism version 7 and R (ggalluvial package).

## Results

Long-term clinical and immunoglobulin data were available for 142 patients with hypogammaglobulinemia. Mild hypogammaglobulinemia was recorded in 40/142 (28.2%), moderate in 66/142 (46.5%) and severe in 36/142 (25.4%) patients. Mean follow-up was 97.2 months; and was longer in lower nadir IgG subgroups (**Table 1**). Patients with more severe hypogammaglobulinemia were younger at diagnosis and first RTX (**Table 1**). AAV was the most common indication for RTX (71%) and most patients received RTX for the management of relapsing (25%) or refractory (69%) disease (**Table 1**). There was no difference in indication for RTX (new, relapsing, or refractory disease), or by diagnosis (p =0.27, data not shown) by subgroup. Seventy one percent were female, with a greater proportion in patients with moderate and severe hypogammaglobulinemia (**Table 1**).

**Table 1:** Patient characteristics.

	All (n = 142)	Mild (n = 40)	Moderate (n = 66)	Severe (n = 36)
Total follow-up (months)	97.2 ± 36.4	87.5 ± 33.7	95.7 ± 34.1	110.6 ± 40.1
Age (years)	45.2 ± 17.6	47.9 ± 17.7	47.6 ± 16.7	37.4 ± 17.2
Age at first RTX (years)	51.4 ± 16.5	55.8 ± 15.8	52.4 ± 15.2	44.2 ± 17.7
Disease duration (months)	43.1 [13.2 – 101.7]	63.2 [10.8 – 159.2]	31.7 [11.7 – 76.8]	56.0 [19.4 – 97.7]
Female	101/142 (71)	21/40 (53)	50/66 (76)	30/36 (83)
Diagnosis				
AAV	101/142 (71)	30/40 (75)	48/66 (73)	23/36 (64)
GPA	69/101 (68)	21/30 (70)	34/48 (71)	14/23 (61)
MPA	15/101 (15)	4/30 (13)	6/48 (13)	5/23 (22)
EGPA	17/101 (17)	5/30 (17)	8/48 (17)	4/23 (17)
SLE	18/142 (13)	5/40 (13)	6/66 (9)	7/36 (19)
Other*	23/142 (16)	5/40 (13)	12/66 (18)	6/36 (17)
Disease state				
New	8/140 (6)	1/39 (3)	5/66 (8)	2/35 (6)
Relapse	35/140 (25)	10/39 (26)	16/66 (24)	9/35 (26)
Refractory	97/140 (69)	28/39 (72)	45/66 (68)	24/35 (69)

Mild: nadir IgG 5 to < 7 g/L, Moderate: nadir IgG 3 to < 5 g/L, Severe: nadir IgG < 3 g/L.

*other: Undifferentiated connective tissue disorder (4), Neuromyelitis optica (3), Undifferentiated vasculitis (2), Behcet’s syndrome (2), polychondritis (2), mixed connective tissue disease (2), IgA vasculitis (1), cryoglobulinemic vasculitis (1), polyartertitis nodosa (1), Cogan’s syndrome (1), Takayasu arteritis (1), myasthenia gravis (1), cryoglobulinemic vasculitis (1).

AAV, ANCA-associated vasculitis; GPA, granulomatosis with polyangiitis; MPA, microscopic polyangiitis; EGPA, eosinophilic granulomatosis with polyangiitis; SLE, systemic lupus erythematosus.

Mean ± standard deviation, median [interquartile range].

### Immunosuppression and Development of Hypogammaglobulinemia

Exposure to mycophenolate mofetil prior to RTX was more common in patients with moderate or severe hypogammaglobulinemia (**Table 2**). Prednisolone use at 12 and 24 months following RTX commencement were associated with lower nadir IgG (**Table 2**). Cumulative RTX dose and prior exposure to other immunosuppressive agents were not associated with a lower nadir IgG (**Table 2**).

**Table 2:** Use of immunosuppressive agents in patients with hypogammaglobulinemia.

	All (n = 142)	Mild (n = 40)	Moderate (n = 66)	Severe (n = 36)	p
Cumulative RTX (g)	9.0 ± 5.1	8.5 ± 4.7	9.8 ± 5.6	8.1 ± 4.4	0.23
**Pre-RTX immunosuppression**					
Cyclophosphamide	107/142 (75)	29/40 (73)	49/65 (75)	28/36 (78)	0.79
Cumulative cyclophosphamide dose (g)	12.0 [6.0 – 26.0]	12.0 [5.8 – 27.8]	11.5 [6.0 – 17.3]	11.0 [5.7 – 27.0]	0.91
Azathioprine	88/141 (62)	27/40 (68)	39/65 (60)	22/36 (61)	0.54
Mycophenolate mofetil	94/141 (67)	25/40 (63)	39/65 (60)	30/36 (83)	0.05
Methotrexate	36/141 (26)	10/40 (25)	20/65 (31)	6/36 (17)	0.42
Intravenous immunoglobulin	22/141 (16)	7/40 (18)	8/65 (12)	7/36 (19)	0.86
Plasma exchange	16/141 (11)	4/40 (10)	5/65 (8)	7/36 (19)	0.27
No. immunosuppressive medications	3.0 [2.0 – 4.0]	3.0 [2.0 – 3.0]	3.0 [2.0 – 3.0]	3.0 [2.0 – 4.0]	0.49
**Concurrent immunosuppression**					
Cyclophosphamide	25/141 (18)	6/40 (15)	13/66 (20)	6/35 (17)	0.77
Mycophenolate mofetil	21/141 (15)	5/40 (13)	9/66 (14)	7/35 (20)	0.39
Plasma exchange	10/141 (7)	4/40 (10)	3/66 (5)	3/35 (9)	0.79
**Post-RTX immunosuppression**					
Cyclophosphamide	20/142 (14)	6/40 (15)	8/66 (12)	6/36 (17)	0.87
Mycophenolate mofetil	27/142 (19)	5/40 (13)	14/66 (21)	8/36 (22)	0.25
No. immunosuppressive medications	0.0 [0.0 – 1.0]	0.5 [0.0 – 1.0]	0.0 [0.0 – 1.0]	1.0 [0.0 – 1.8]	0.44
**Prednisolone**					
Baseline	115/121 (95)	36/38 (95)	53/55 (96)	26/28 (93)	0.82
6 months	120/133 (90)	31/39 (79)	61/63 (97)	28/31 (90)	0.15
12 months	113/137 (82)	27/39 (69)	56/64 (88)	30/34 (88)	0.04
24 months	98/133 (74)	22/37 (59)	48/62 (77)	28/34 (82)	0.03

Mild: nadir IgG 5 to < 7 g/L, Moderate: nadir IgG 3 to < 5 g/L, Severe: nadir IgG < 3 g/L.

RTX, rituximab. Proportion (%), median [interquartile range].

### Immunoglobulin Levels Over Long-Term Follow-Up

Baseline values were often collected after commencement of glucocorticoids; mean IgG at baseline was 7.45 (standard deviation (SD) 3.1), mean baseline IgM was 0.8 (SD0.5) and mean baseline IgA was 1.6 (0.8).

**Figure 1:**
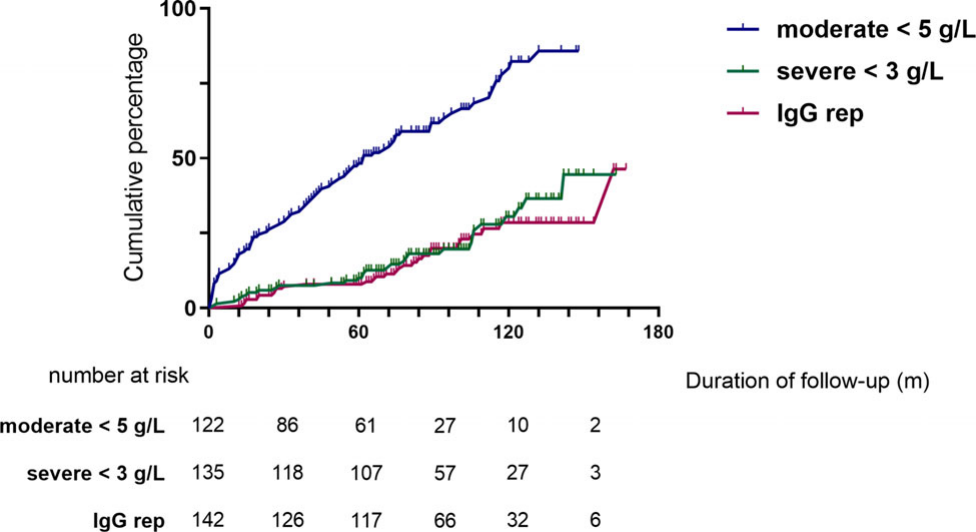
Cumulative incidence of hypogammaglobulinemia and immunoglobulin replacement therapy commencement during follow-up.

Moderate (IgG <5 g/L) and severe (IgG <3 g/L) hypogammaglobulinemia and use of immunoglobulin replacement therapy was increasingly observed with longer follow-up (**Figure 1**). Median time to moderate hypogammaglobulinemia was 22.5 months [IQR 3.0 to 61.5] and to severe hypogammaglobulinemia was 24.5 months [IQR 4.0 to 80.8].

**Figure 2:**
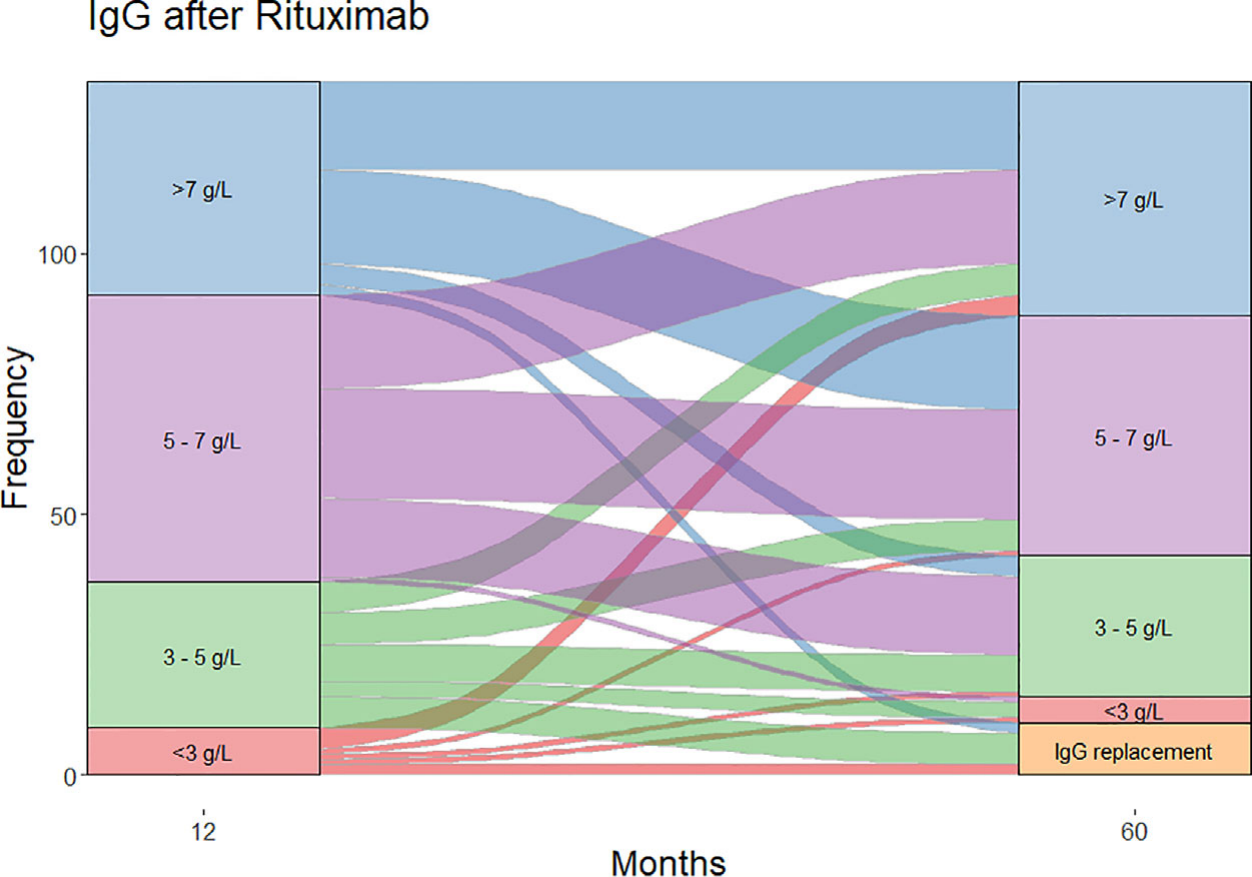
Change in IgG strata between month 12 and month 60 of follow-up.

Of the patients who were followed up to 60 months post-RTX (n=124), substantial change was observed in IgG levels over time (**Figure 2**). In patients with moderate hypogammaglobulinemia within the first 12 months of RTX administration, 17/37 (45%) patients recovered to an IgG ≥5 g/L without the need for immunoglobulin replacement therapy at 60 months. A further 8/37 (22%) had commenced immunoglobulin replacement therapy, and the remaining 12/37 (32%) remained hypogammaglobulinemia with an IgG <5 g/L at 60 months.

In a multivariable logistic regression model, cyclophosphamide use prior to RTX, lower nadir IgG in the first 12 months, prednisolone use at 12 months following RTX, and female sex were associated with an increased likelihood of moderate hypogammaglobulinemia and/or requiring immunoglobulin replacement 60 months after RTX commencement. This model was additionally adjusted for age at RTX commencement, mycophenolate use prior to RTX and total cumulative RTX (**Table 3**).

**Table 3:** IgG < 5 g/L or immunoglobulin replacement at 60 months.

	Model 1 OR (95% CI)	p- value	Model 2 OR (95% CI)	p-value
Age at RTX commencement	0.98 (0.95 – 1.01)	0.21	0.97 (0.94 – 1.01)	0.10
Female	7.56 (1.88 – 30.48)	0.004	8.57 (2.07 – 35.43)	0.008
Pre-RTX cyclophosphamide	3.31 (1.00 – 10.96)	0.05	3.60 (1.03 – 12.53)	0.04
Pre-RTX mycophenolate	2.16 (0.75 – 6.26)	0.16	2.04 (0.70 – 5.95)	0.20
Nadir IgG (0 – 12 m)	0.67 (0.50 – 0.90)	0.008	0.68 (0.51 – 0.90)	0.008
Prednisolone use at 12 m	6.19 (1.12 – 33.31)	0.03	7.48 (1.28 – 43.55)	0.03
Total cumulative RTX	0.91 (0.81 – 1.02)	0.09	0.91 (0.81 – 1.02)	0.11
Nadir IgM (0 – 12 m)	–		0.12 (0.01 – 1.05)	0.06

RTX, rituximab; Ig immunoglobulin; m, month, OR, odds ratio, CI, confidence interval.

Cumulative RTX dose was not associated with a greater likelihood of moderate/severe hypogammaglobulinemia or requiring immunoglobulin replacement therapy 60 months after RTX commencement. The inclusion of disease duration prior to RTX and number of immunosuppressive agents used post-RTX did not improve model fit or alter overall interpretation. A model inclusive of nadir IgM values within the first 12 months improved model fit, with no change in interpretation (**Table 3**).

### Hypogammaglobulinemia and Infection

**Figure 3:**
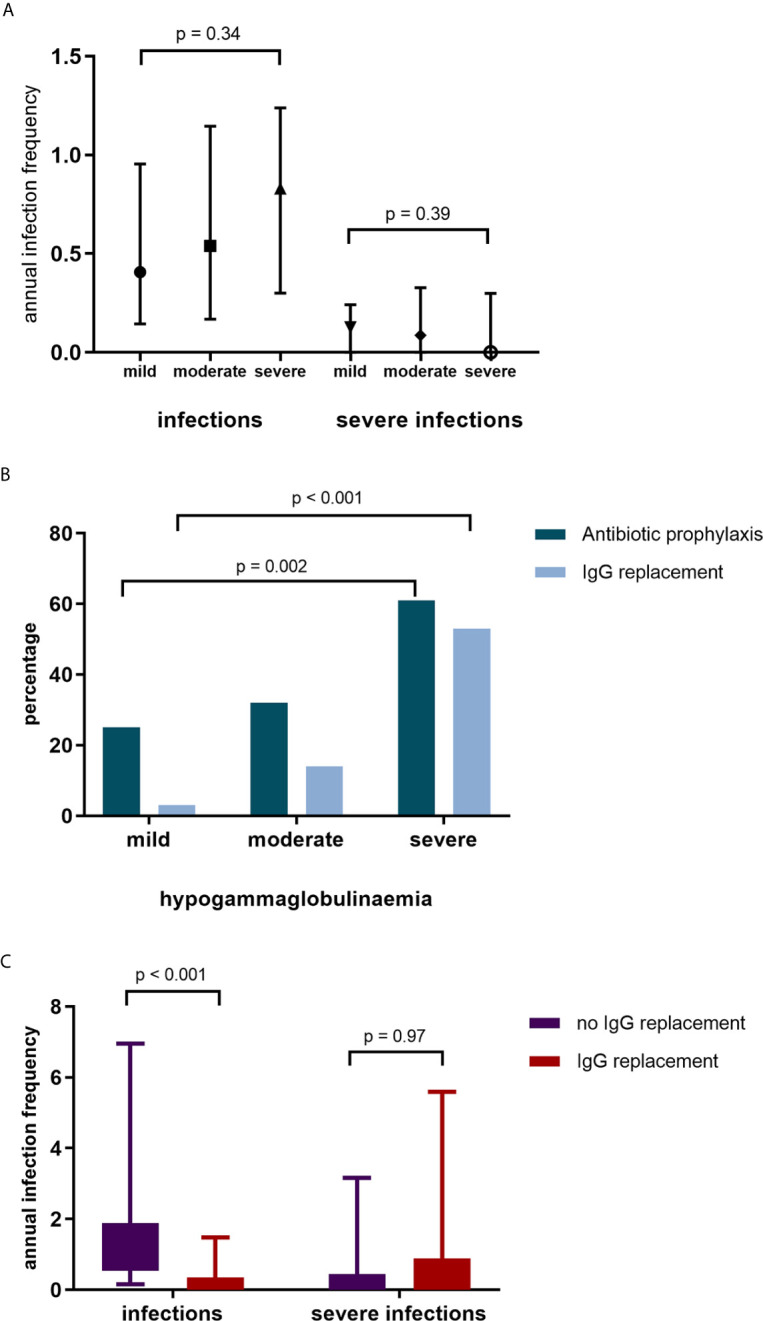
**(A)** Annual infection and severe infection rate by IgG subgroup. **(B)** Commencement of antibiotic (Abx) prophylaxis and IgG replacement by IgG group. **(C)** Infection and severe infection rates in patients without IgG replacement and during IgG replacement.

Overall, infection rates were low. Severe and non-severe infections predominantly involved the respiratory tract (65% and 58% respectively). There were no differences in infection rates between patients with mild, moderate, and severe hypogammaglobulinemia (**Figure 3A**). A subset of patients, however, were referred for further assessment and/or commenced prophylactic therapy due to recurrent infections.

Peripheral blood immunophenotyping was available in 30 patients at the time of Clinical Immunology assessment; CD19+ lymphocytes were detectable in 11 (37%). Where sufficient B cells were identified in 8 of these patients (7 with AAV and 1 with SLE), further B cell subset analyses were performed. This revealed a pattern of high naïve (IgM^+^IgD^+^CD27^-)^ and low switched memory (IgM^-^IgD^-^CD27^+)^ B cells in all patients (**Supplementary Table 1**).

Pneumococcal antibody titers were available in 28 patients with recurrent infection, with only 9 having protective antibody titers to at least 7 of the 13 serotypes tested. In those who went on to receive immunoglobulin replacement therapy, only 4 of 18 patients tested (22%) had protective pneumococcal antibody levels, and a post-vaccination response was demonstrated in only 1/9 (11%) recorded.

### Antibiotic Prophylaxis and Immunoglobulin Replacement Therapy

Antibiotic prophylaxis was initiated in 53 (37%) of patients; greater antibiotic prophylaxis use was observed in patients with moderate and severe hypogammaglobulinemia (**Figure 3B**). Of the patients who commenced antibiotic prophylaxis, 42 (79%) were AAV patients, 6 (11%) had SLE and 5 (9%) other autoimmune conditions. Immunoglobulin replacement therapy was initiated in 27/53 (51%) patients who had commenced antibiotic prophylaxis.

Immunoglobulin replacement therapy was commenced in 29 patients; with mild hypogammaglobulinemia in 1 (3%) patient, moderate hypogammaglobulinemia in 9 patients (31%) and severe hypogammaglobulinemia in 19 patients (66%). Of the patients commencing immunoglobulin replacement therapy, 21 (72%) had a diagnosis of AAV, 4 (14%) SLE and 4 (14%) other autoimmune diseases. Immunoglobulin replacement therapy was commenced a median of 71 months after first RTX. In patients commencing immunoglobulin replacement therapy, infections reduced (median [IQR] 1.02 infections/year [0.54 – 1.88] to 0.13 infections/year [0.00 – 0.35], p <0.001, **Figure 3C**). Annual severe infection rates were not reduced during immunoglobulin replacement therapy in these patients. After removal of two outliers with recurrent respiratory tract infections requiring antibiotics, there remained no difference in severe infection rates.

At the time of data collection or last recorded follow-up, 20 of 29 patients were continuing to receive immunoglobulin replacement therapy, 4 had died and 5 had ceased immunoglobulin replacement therapy. Of the four who died, the causes of death were respiratory sepsis in a patient with AAV, decompensated liver disease and pneumonia in a patient with IgA vasculitis, refractory vasculitis in a patient with AAV and was unknown in a patient with AAV. Of the five who had ceased immunoglobulin replacement therapy, 2 were intolerant and 3 were weaned off immunoglobulin replacement therapy without recurrent infection; 1 subsequently recommenced immunoglobulin replacement therapy owing to recurrent infection, 1 has had IgG recovery to normal levels (>7 g/L), and 3 have remained off immunoglobulin replacement therapy with stable IgG levels <5 g/L.

## Discussion

We report on 142 patients with multi-system autoimmune disease with RTX associated hypogammaglobulinemia, their long-term outcomes and response to immunoglobulin replacement therapy. Overall, 102/142 (72%) had moderate hypogammaglobulinemia and 36/142 patients (25%) severe hypogammaglobulinemia. Factors associated with lower nadir IgG levels were prior mycophenolate use and prednisolone use 12 and 24 months after RTX initiation. Prior cyclophosphamide, prednisolone at 12 months after RTX initiation, nadir IgG in the first 12 months of RTX commencement and female sex were associated with an increased likelihood of moderate/severe hypogammaglobulinemia and/or immunoglobulin replacement therapy use 60 months post-RTX commencement. Antibiotic prophylaxis was used in 53/142 (37%) patients and immunoglobulin replacement therapy commenced in 29/142 (20%) in whom infection rates but not severe infection rates were reduced.

The majority of patients included in this study had refractory SLE and AAV. There is substantial consistent evidence that RTX is beneficial in patients with AAV in both induction and maintenance of remission (8–10). Although data for RTX in SLE has been mixed, observational studies have demonstrated benefit (11, 12). Although hypogammaglobulinemia has been identified in multiple observational studies, the occurrence of hypogammaglobulinemia in this cohort is higher than previous estimates (7, 13–16). This cohort had a longer duration of follow-up, with nadir IgG levels occurring many months or years after commencing RTX therapy. Mean follow-up was 8 years, compared with up to an average follow-up of 4 years in other studies (2, 7, 13–18).

The rate of hypogammaglobulinemia may also be influenced by diagnosis. Although most patients in this study had AAV, other studies of hypogammaglobulinemia have included greater proportions of patients with RA (not included in this study) and SLE (13% of this cohort) (1, 16, 18). Thiel and colleagues have demonstrated delayed B cell recovery following RTX in patients with AAV compared with RA and SLE, suggesting a distinct underlying or acquired B cell dysfunction in these patients (19).

Notably, cumulative RTX doses are higher in this study than other reports (6, 7, 15, 17). This is likely influenced by multiple factors including the duration of follow-up and high proportion of patients with longstanding relapsing or refractory disease in this cohort. An association between cumulative RTX and hypogammaglobulinemia has previously been postulated (6), but not identified in other studies (2, 4, 7). In this study, there was no difference in cumulative RTX dose across the subgroups and was not associated with greater likelihood of moderate/severe hypogammaglobulinemia or requiring immunoglobulin replacement therapy at 60 months in an adjusted logistic regression model.

The impact of other immunosuppressive agents used prior to, in conjunction with or after RTX in the development of hypogammaglobulinemia has been difficult to delineate. Of note, mean baseline immunoglobulin levels were low-normal at baseline. In this study, mycophenolate and cyclophosphamide were the most common non-glucocorticoid immunosuppressive agents used. In the multivariable logistic regression model accounting for age, sex and prednisolone use post-RTX, prior cyclophosphamide, but not mycophenolate use increased the likelihood of moderate or severe hypogammaglobulinemia 60 months after RTX initiation. Venhoff and colleagues observed prolonged B cell depletion in patients who received RTX after previous cyclophosphamide use compared with RTX alone (20). In this study, 54% of patients who had received prior cyclophosphamide developed hypogammaglobulinemia, compared with 21% who received RTX alone.

Glucocorticoids alone have also been implicated in the development of hypogammaglobulinemia, and the impact of prolonged or greater glucocorticoid use in conjunction with RTX or other immunosuppressive agents on immunoglobulin levels requires further study (21). In this cohort, prednisolone use at 12 and 24 months were associated with lower nadir immunoglobulin levels. In the multivariable model examining outcomes at 5 years, prednisolone use at 12 months was associated with increased likelihood of moderate or severe hypogammaglobulinemia and/or immunoglobulin replacement. The use of prednisolone at 12 months was observed in 82.5% of patients, reflective of clinical practice in patients with historically more difficult to control, longstanding disease. Ongoing efforts to minimize glucocorticoid exposure remain important to the chronic management of these patients.

Of interest, there were more female patients were more likely to have more likely to have moderate/severe hypogammaglobulinemia and/or have commenced immunoglobulin replacement therapy at 60 months. Cross sectional studies suggest that immunoglobulin levels decline with age, with limited differences between males and females in adult age ranges (22–24). In post-hoc analyses of a trial evaluating induction therapy in AAV, female patients receiving RTX had higher serum RTX levels compared with males despite using body surface area dosing (25). Importantly, however, although higher serum levels of RTX were associated with a longer time to B cell repopulation, this was not associated with fewer relapses up to 18 months of follow-up. This association requires further assessment in larger cohorts and could have implications for dosing based on sex if confirmed.

Infections remain the key concern in patients with hypogammaglobulinemia. In a mixed cohort of patients receiving RTX for cancer (77.7%) and rheumatologic conditions (27.7%), severe infection rates were greater in patients with hypogammaglobulinemia (26). This was observed in early follow-up 12 months after RTX by MD Yusof and colleagues who in a mixed cohort of patients with autoimmune rheumatic diseases, identified an increased likelihood of severe infections in patients with hypogammaglobulinemia (16).

The use of immunoglobulin replacement therapy in patients with hypogammaglobulinemia associated with immunosuppression is extrapolated from experience in the management of the heterogenous group of patients with CVID. Both groups share a predisposition to infection, hypogammaglobulinemia, and impaired vaccination responses. In CVID, a reduction in respiratory tract infections has been demonstrated in small cohorts after commencement of immunoglobulin replacement therapy (27–29). The efficacy of immunoglobulin replacement therapy in patients with hypogammaglobulinemia and hematological malignancies has also been demonstrated in small cohorts (30). We observed a reduction in infection rates after initiation of immunoglobulin replacement therapy, supporting the efficacy of immunoglobulin replacement therapy in this population of patients with systemic autoimmune disease.

Importantly, despite the reduction in infections requiring antimicrobial therapy, the same benefit was not observed for severe infections. The majority of severe infections in these patients were respiratory tract infections; in this patient population, disease related airways damage and colonization of the respiratory tract commonly contribute to chronic and recurrent infections, which may not be mitigated by immunoglobulin replacement. Age and other comorbidities may additionally influence infections in this cohort of patients with refractory and relapsing disease.

Given the patient and health care burdens of ongoing immunoglobulin replacement therapy, and increasing concerns regarding supply of this limited resource, trials of immunoglobulin replacement therapy cessation are considered. However, the most appropriate approach to this remains unknown. Recovery of immunoglobulin levels was observed in several individuals in longer term follow-up. In this single center study, of the 29 patients who commenced immunoglobulin replacement therapy, it was successfully ceased in 4 of the 5 patients in whom this was attempted. Although a very limited experience is presented in this study, it highlights the possibility of cessation of immunoglobulin replacement.

Again, albeit in small numbers, the pattern of high naïve and low switched memory B cells observed in a subset of these patients with hypogammaglobulinemia despite B cell repopulation warrants further investigation. Although a possible treatment effect, this could be representative of an associated underlying B cell dysfunction, which has been suggested in the associations between CVID and autoimmunity (31, 32).

Limitations of this study include the retrospective design, introducing selection bias in choice of treatments and total doses. Long-term follow-up in patients who have difficult to control rare autoimmune disease has inherent challenges. Though missing data, particularly for infection and severe infections, which were often not culture proven, is an important limitation, this group of patients typically have close clinical review focusing on infections, an important contributor to morbidity in this group of patients. The lack of control group for comparisons of infection and severe infection rates is a limitation to this study. Some studies have drawn comparisons between cyclophosphamide and rituximab treated patients. As refractory disease or disease relapse are common in long term follow-up, overlap of medications are common, and limit comparisons between groups.

In this study evaluating long-term outcomes of patients with RTX associated hypogammaglobulinemia, we have observed clinically significant hypogammaglobulinemia in a high proportion of patients, and an increasing incidence of hypogammaglobulinemia over time. The rates observed highlight the need for ongoing immunoglobulin monitoring in patients who have previously or continue to receive RTX. The use of prior immunosuppressive therapies, prolonged glucocorticoid use and female gender were associated with hypogammaglobulinemia long-term. Additionally, a reduction in infection in those receiving immunoglobulin replacement therapy for recurrent infection, provides evidence of its efficacy in this population of immunodeficient individuals. The risks and consequences of hypogammaglobulinemia should be considered with RTX therapy in multi-system autoimmune disease.

## Data Availability Statement

The raw data supporting the conclusions of this article will be made available by the authors, without undue reservation upon reasonable request.

## Ethics Statement

In accordance with the UK National Health Service Research Ethics Committee guidelines, ethics approval was not required as this work comprises anonymous retrospective data and all treatment decisions were made prior to our evaluation.

## Author Contributions

JT, RS, DK, and DJ contributed to conception and design of the study. JT and SG extracted data. JT performed the statistical analysis. All authors contributed to interpretation of results. JT wrote the first draft of the manuscript. All authors contributed to the article and approved the submitted version.

## Conflict of Interest

JT reports grants from Arthritis Australia (funded by Australian Rheumatology Association and Roche) and National Health and Medical Research Council during the conduct of this study. DK reports other support from CSL Behring, Shire/Takeda, Charities Fund Addenbrookes Hospital Cambridge and Grifols outside the submitted work, and membership of Immunoglobulin Demand Management Assessment Panel for National Health Service UK, membership of Clinical Reference Group for Immunology and Allergy National Health Service, England since 2019. AM reports personal fees and other support from CSL Behring, and other support from Takeda outside submitted work. DJ reports grants from Roche/Genentech during the conduct of the study, personal fees from Astra-Zeneca, Aurinia, and Boehringer, grants and personal fees from Chemocentryx, grants and personal fees from GSK, and grants from Sanofi outside the submitted work.

The remaining authors declare that the research was conducted in the absence of any commercial or financial relationships that could be construed as a potential conflict of interest.

The handling editor declared a past co-authorship with one of the authors, DJ.
