# Supplementary material for: Rituximab Associated Hypogammaglobulinemia in Autoimmune Disease
Source: Front Immunol. 2021 May 12;12:671503. doi: 10.3389/fimmu.2021.671503 (PMC8149951; doi:10.3389/fimmu.2021.671503)
Supplement: Supplementary file 1 [file Table_1.docx]

| Patient | Diagnosis | Lymphocyte count  (x 10^9^/L) | CD19  (x 10^9^/L) | Naïve B cells  IgM+/IgD+/CD27-  (42.6 – 82.3%) | Non-switched memory B cells  IgM+/IgD+/CD27+ | Switched memory B cells  IgM-/IgD-/CD27+  (6.5 – 29.1%) |
| --- | --- | --- | --- | --- | --- | --- |
|  | | | | | | |
| 1 | AAV | 6.41 | 0.12 | 97.10 | 2.20 | 0.30 |
| 2 | AAV | 2.42 | 0.75 | 96.80 | 2.20 | 0.60 |
| 3 | AAV | 2.97 | 0.33 | 94.70 | 4.40 | 0.30 |
| 4 | AAV | 0.91 | 0.15 | 90.60 | 7.70 | 1.10 |
| 5 | AAV | 1.36 | 0.15 | 97.50 | 2.30 | 0.00 |
| 6 | AAV | 0.84 | 0.08 | 97.0 | 1.80 | 0.20 |
| 7 | AAV | 1.21 | 0.13 | 93.50 | 3.80 | 0.80 |
| 8 | SLE | 1.25 | 0.13 | 97.50 | 1.60 | 0.30 |

Supplementary Table 1

AAV ANCA associated vasculitis, SLE Systemic lupus erythematosus
